# Impact of Display Pixel–Aperture Ratio on Perceived Roughness, Glossiness, and Transparency

**DOI:** 10.3390/jimaging11040118

**Published:** 2025-04-16

**Authors:** Kosei Aketagawa, Midori Tanaka, Takahiko Horiuchi

**Affiliations:** 1 Graduate School of Science and Engineering, Chiba University, Yayoi-cho 1-33, Inage-ku, Chiba 263-8522, Japan; 24wm3210@student.gs.chiba-u.jp; 2 Graduate School of Informatics, Chiba University, Yayoi-cho 1-33, Inage-ku, Chiba 263-8522, Japan; horiuchi@faculty.chiba-u.jp

**Keywords:** pixel–aperture ratio, shitsukan, total appearance, roughness, glossiness, transparency, display technology

## Abstract

Shitsukan, which encompasses the perception of roughness, glossiness, and transparency/translucency, represents the comprehensive visual appearance of objects and plays a crucial role in accurate reproduction across various fields, including manufacturing and imaging technologies. This study experimentally examines the impact of the pixel–aperture ratio on the perception of roughness, glossiness, and transparency. A visual evaluation experiment was conducted using natural images presented on stimuli with pixel–aperture ratios of 100% and 6%, employing an RGB sub-pixel array. The results demonstrated that the pixel–aperture ratio significantly affects the perception of glossiness and transparency, with the 100% pixel–aperture ratio producing a statistically significant effect compared to the 6% condition. However, roughness perception varied substantially among the observers, and no statistically significant effect was observed. Nonetheless, when comparing two observer clusters identified through clustering analysis, the cluster favoring the 100% pixel–aperture ratio exhibited “Huge” effect sizes for all perceptual attributes. Additionally, the findings indicate that the degree of influence of pixel–aperture ratio on glossiness and transparency is not constant and can vary depending on individual observer differences and image characteristics.

## 1. Introduction

Humans evaluate the appearance and quality of objects based on visual cues that arise from the interaction between the optical properties of an object’s surface and the surrounding lighting conditions [1]. Shitsukan, which captures an object’s surface characteristics, plays a crucial role in these assessments. The term shitsukan is a Japanese word referring to the comprehensive perception derived from physical stimuli processed through multiple sensory modalities. This integrated perception includes higher-order psychophysical elements and encompasses visual attributes such as roughness, glossiness, and transparency perception. Research on shitsukan has advanced in recent years across multiple disciplines, including information engineering, psychophysics, and neuroscience [2]. The importance of accurately reproducing and conveying shitsukan has gained increasing recognition. Studies in this field have focused on evaluating objects’ visual appearance and have explored perceptual elements such as glossiness and roughness through psychophysical experiments and the incorporation of tactile feedback.

For instance, roughness perception is a fundamental aspect of shitsukan that is directly linked to an object’s surface condition and is influenced by both visual and tactile modalities. Bergmann Tiest et al. [3] demonstrated the interaction between tactile and visual input in roughness perception, showing that physically measured roughness does not always align with subjective perception. Moreover, Fleming [4] noted that roughness perception is not merely a direct evaluation of physical properties but is shaped through a statistical generative model. The visual system synthesizes multiple cues, such as spatial frequency features and light reflection patterns, while incorporating prior knowledge and expectations. Additionally, research has shown that variations in lighting and viewpoint can lead to differences in roughness impressions for the same physical stimulus. Similarly, in glossiness perception, Motoyoshi et al. [5] investigated the relationship between image statistics and gloss perception, demonstrating that the spatial distribution and intensity of reflected light play a pivotal role in shaping visual glossiness. Transparency perception, in contrast, is influenced by multiple factors, including light refraction, scattering, and contrast against the background [6]. Fleming et al. further clarified that the perceived transparency of materials is affected not only by optical characteristics but also by objects’ shape and edge structure [7]. These findings collectively suggest that shitsukan perception arises from the interplay between an object’s physical properties and the observer’s cognitive processes.

Meanwhile, with advancements in display technology, the demand for accurate shitsukan reproduction in screen-displayed images has been increasing. Our previous studies experimentally examined the effects of different sub-pixel arrays (RGB, RGBW, and PenTile RGBG) on the perception of roughness, glossiness, and transparency, demonstrating that sub-pixel array structures significantly influence shitsukan perception [8,9]. Furthermore, we investigated the impact of the pixel–aperture ratio and experimentally confirmed that variations in this ratio lead to differences in perceived resolution [10]. However, the impact of pixel–aperture ratio on shitsukan perception has not been sufficiently explored. Recently, OLED displays have been widely adopted due to their high contrast and slim profile, making them valuable for various applications. Meanwhile, in pursuit of further performance enhancements, mini-LED and micro-LED displays, which offer high energy efficiency, brightness, color purity, and longevity, have been commercialized [11]. These displays consist of extremely fine LED elements, with mini-LEDs measuring 100–200 µm and micro-LEDs measuring below 100 µm. Compared to conventional LCDs and OLEDs, mini-LED and micro-LED displays exhibit a significantly lower pixel–aperture ratio [12]. Although these technologies are now widely available in the market, research on their impact on shitsukan perception remains insufficient. Therefore, clarifying how variations in pixel–aperture ratio affect the perception of roughness, glossiness, and transparency and providing insights into pixel design for improved shitsukan reproduction is a critical issue.

Given this context, this study aims to investigate the influence of different pixel–aperture ratios on the perception of roughness, glossiness, and transparency in displayed images. A visual evaluation experiment was conducted using 23 types of natural images presented with two distinct pixel structures (100% and 6% pixel–aperture ratios) to analyze their effects on the three shitsukan attributes. Furthermore, based on the experimental results, we assess statistical significance through effect size analysis, perform observer clustering, and examine image characteristics to investigate individual observer tendencies and the influence of image properties on shitsukan perception.

## 2. Paired Stimulus Experiment

In this experiment, a visual evaluation of shitsukan perception was conducted using stimuli with two different pixel–aperture ratios across 23 types of natural images to analyze how variations in pixel–aperture ratio influence shitsukan perception in displayed natural images.

### 2.1. Experimental Stimuli

**Figure 1:**
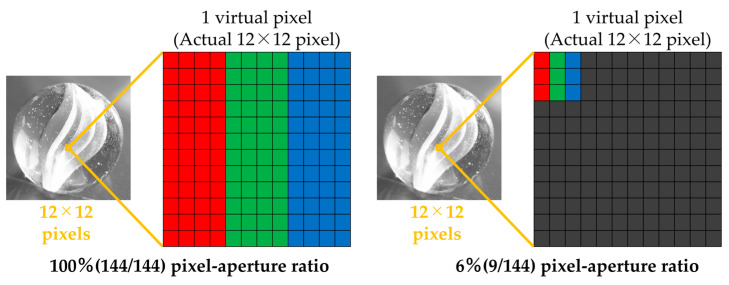
Construction of the virtual pixel structure for experimental stimuli.

The experimental stimuli were generated using a simulation method based on previous studies [8,9,10] to create stimuli with different pixel–aperture ratios. The stimulus creation process is described as follows. The display used in this experiment (ColorEdge PROMINENCE CG3146, EIZO) features an RGB sub-pixel array. Since obtaining multiple displays that differ solely in pixel structure is difficult, we simulated two types of pixel structures by considering a 12 × 12 px region of the display as a single virtual pixel. Figure 1 illustrates the two pixel–aperture configurations used in the experiment (100% and 6% pixel–aperture ratios). The sub-pixel array was set as an RGB sub-pixel array, which is widely adopted in general displays. To standardize the virtual pixel width across different pixel structures, the virtual sub-pixel widths were set as follows: 4 × 12 px for the 100% pixel–aperture ratio and 1 × 3 px for the 6% pixel–aperture ratio. Furthermore, to ensure that brightness differences between stimuli did not influence the evaluation, a spectroradiometer (CS-2000, Konica Minolta, Inc.) was used to adjust the pixel values so that the average luminance of the experimental stimuli remained equal across the two pixel–aperture conditions. The maximum luminance of the experimental stimuli was calibrated so that white consistently measured 21.2 cd/m^2^. Each 12 × 12 px area created through this procedure was treated as a single virtual pixel. When evaluating experimental stimuli with two different pixel structures, a viewing distance of 12 times a single real pixel was required to properly observe one virtual pixel. Section 3.1 presents the modulation transfer function (MTF) analysis of the generated stimuli as part of their physical characterization.

**Figure 2:**
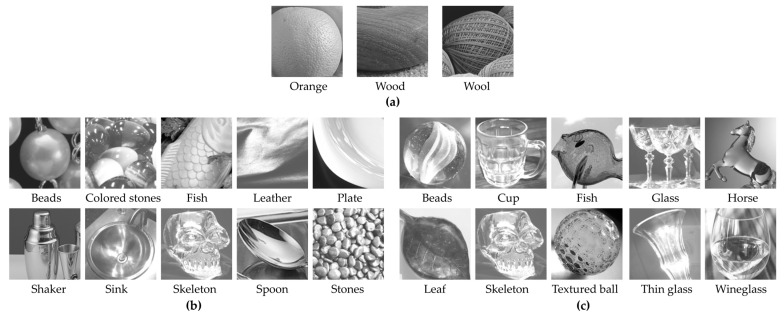
List of image content used as experimental stimuli: (**a**) roughness; (**b**) glossiness; (**c**) transparency.

Next, the natural images used in the experiment are described. A total of 23 natural images were selected from standard image databases (SCID, SHIPP) and the Flickr Material Database [13]. These images were cropped to 150 × 150 px, ensuring that sufficient visual information was retained for shitsukan evaluation. In order to clearly evaluate the effect of the pixel–aperture ratio on shitsukan perception, the images were converted to grayscale using the NTSC formula. Our previous study reported that color affects shitsukan impressions, particularly showing that perceptions of qualities such as the “glossiness” of paper and the “prettiness” of rubber differ between color and grayscale images [14]. This result suggests that color has a significant effect on shitsukan evaluation. Therefore, in this study, we used grayscale images to mask the effects of color contrast and color perception. The experimental stimuli were then created by magnifying each real pixel in the cropped images (150 × 150 px) by a factor of 12 × 12, resulting in final stimuli of 1800 × 1800 px (150 × 150 virtual pixels) on the actual display. Figure 2 shows examples of the three types of natural images used for evaluating roughness perception, glossiness perception, and transparency perception. The roughness perception stimuli included images of three different materials, while the glossiness perception stimuli consisted of various gloss types based on Hunter’s six gloss categories [15]. The transparency perception stimuli featured transparent glass objects.

### 2.2. Experimental Procedure

This experiment presented stimulus pairs, simulated with different pixel structures, on the left and right sides of a black background on a display. Observers evaluated which stimulus exhibited a stronger shitsukan perception for three shitsukan attributes—roughness perception, glossiness, and transparency perception—using the two-alternative forced-choice (2AFC) method. To ensure consistency in evaluation across observers, the three attribute terms used in the evaluation—roughness, glossiness, and transparency—were clearly defined and explained in simple, easy-to-understand language prior to the evaluation sessions. Observers received the following verbal instructions, and we confirmed their understanding before the experiments began to prevent any ambiguity or misinterpretation.

- Roughness: “Please select the stimulus that appears rougher, based on the perceived roughness and bumps on the object surface. Note that a low image resolution does not necessarily indicate surface roughness. Please focus on the surface texture of the object itself, not the sharpness or clarity of the image”.
- Glossiness: “Please select the stimulus that appears glossier. Glossiness refers to the visual impression of the reflectivity—how shiny or lustrous the object appears”.
- Transparency: “Please select the stimulus that appears more transparent. All objects shown are made of glass. Transparency refers to the degree to which light passes through the object and how much of the background or surroundings is visible through it. This includes partial transparency, such as that seen in frosted or translucent glass”.

These instructions were designed to minimize subjective interpretation and ensure that all observers evaluated the same perceptual characteristics based on a shared understanding.

To examine the effect of differences in pixel–aperture ratio on shitsukan perception, two stimulus pairs with different pixel structures were created for each image content. The specific stimulus pair combinations were as follows:

100% pixel–aperture ratio vs. 6% pixel–aperture ratio

6% pixel–aperture ratio vs. 100% pixel–aperture ratio

In total, 10 observers with binocular visual acuity equivalent to 20/20 and normal color vision participated in the experiment. In the experiment, observers first underwent a visual acuity test, followed by a 3 min dark adaptation period. After adaptation, stimulus pairs were presented on the left and right sides of a black background in a dark room. Observers were asked to select the stimulus that exhibited a stronger shitsukan perception for each pair. After each response, a black background was displayed for 1 s to minimize the influence of the previously evaluated stimulus. The next stimulus pair was then presented, and the evaluation process was repeated. The viewing distance was set to 30 cycles per degree (cpd), equivalent to 7.01 m, as recommended by ITU-R [16], the radiocommunication sector of the International Telecommunication Union, as the standard viewing distance. To ensure reliability, each stimulus pair was evaluated 8 times. To eliminate the influence of display luminance non-uniformity, the left and right positions of the stimulus pairs were swapped during the presentation. Throughout the experiment, roughness perception was evaluated 48 times per observer (2 pairs × 8 evaluations per pair × 3 stimulus types × 1 viewing distance), while glossiness perception and transparency perception were each evaluated 160 times per observer (2 pairs × 8 evaluations per pair × 10 stimulus types × 1 viewing distance). The order of stimulus presentation was randomized to minimize bias.

## 3. Results and Discussions

This section presents the results of the visual evaluation experiment and analyzes the impact of each pixel structure on shitsukan perception alongside Modulation Transfer Function (MTF) analysis. Additionally, the effects of individual observer response tendencies and image characteristics on the results are examined. Furthermore, since the same natural images and analytical methods were used as in previous studies [8,9]—which investigated the effects of three types of sub-pixel arrays (RGB, RGBW, and PenTile RGBG) on shitsukan perception—comparisons with these studies are also discussed.

### 3.1. MTF Calculation

The Modulation Transfer Function (MTF) represents the magnitude of response to sinusoidal waves of different spatial frequencies and provides an objective and quantitative evaluation of a display’s spatial frequency characteristics. In a previous study [10], the MTF was employed for the analysis and discussion of perceptual resolution. In this study, the MTF was also calculated to account for differences in the physical conditions of pixel–aperture ratios and was analyzed alongside the evaluation results.

Using the MTF enables a quantitative analysis of how variations in spatial frequency characteristics due to pixel structure influence shitsukan perception, making it an effective approach. A higher MTF is expected to result in a clearer image display, leading to an increased perception of shitsukan. As an example, the line spread function LSF(x) of the luminance profile in the vertical projection for the 100% pixel–aperture ratio with an RGB sub-pixel array is given by Equation (1). The horizontal projection follows the same principle.

(1) $$LSF\left(x\right)=2\left(\frac{L_{R}}{L_{RGB}}rect\left(\frac{x+\frac{5}{6}}{\frac{1}{3}}\right)+\frac{L_{G}}{L_{RGB}}rect\left(\frac{x+\frac{3}{6}}{\frac{1}{3}}\right)+\frac{L_{B}}{L_{RGB}}rect\left(\frac{x+\frac{1}{6}}{\frac{1}{3}}\right)\right)$$

Here, rect(·) represents the rectangular function. $L_{R}$, $L_{G}$, and $L_{B}$ denote the luminance of the red, green, and blue sub-pixels, respectively, and $L_{RGB}$ is defined as $L_{RGB}$ = $L_{R}+L_{G}+L_{B}$. The obtained LSF(x) was subjected to a Fourier transform and normalized such that it equals 1 at ξ=0, yielding the following $MTF\left(\xi \right)$ by Equation (2):

(2) $$MTF\left(\xi \right)=\left|sinc\left(\frac{1}{3}\xi \right)\left(\frac{L_{R}}{L_{RGB}}e^{j\frac{5}{3}\pi \xi }+\frac{L_{G}}{L_{RGB}}e^{j\pi \xi }+\frac{L_{B}}{L_{RGB}}e^{j\frac{1}{3}\pi \xi }\right)\right|$$

**Figure 3:**
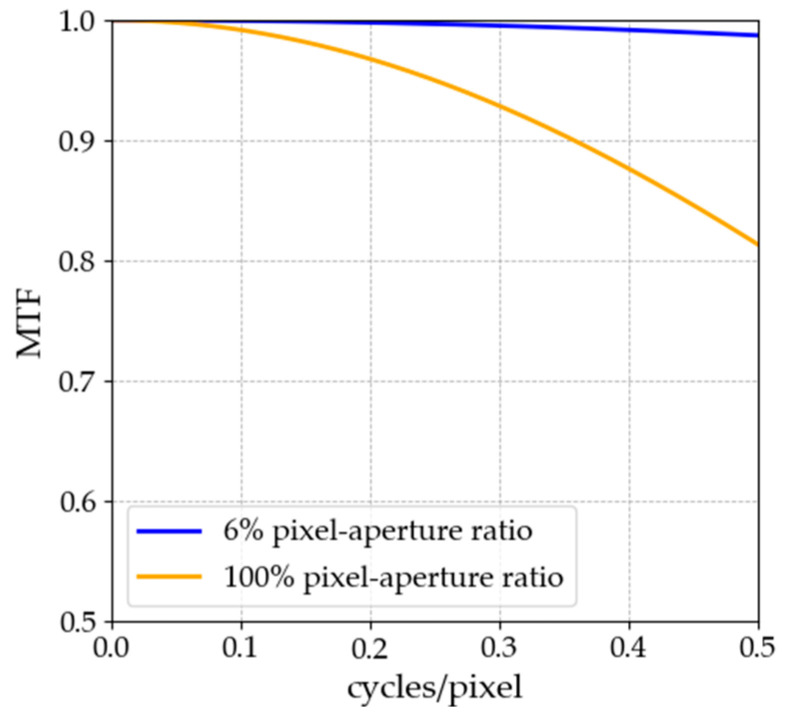
Calculated MTF for both pixel structures.

The MTF of each pixel structure, calculated using the same procedure, is shown in Figure 3. Based on the MTF values, the superiority relationship between the pixel structures was determined as follows: 6% pixel–aperture ratio > 100% pixel–aperture ratio. At the Nyquist frequency of 0.5 cycles per pixel, the difference in MTF values between the two pixel structures (hereafter referred to as the MTF difference) was calculated as follows: MTF difference = MTF of 6% pixel–aperture ratio (0.987)—MTF of 100% pixel–aperture ratio (0.813) = 0.174. Thus, the MTF difference was approximately 17.4%.

### 3.2. Response Rate and Significant Differences

#### 3.2.1. Average Response Rates of 10 Observers

**Figure 4:**
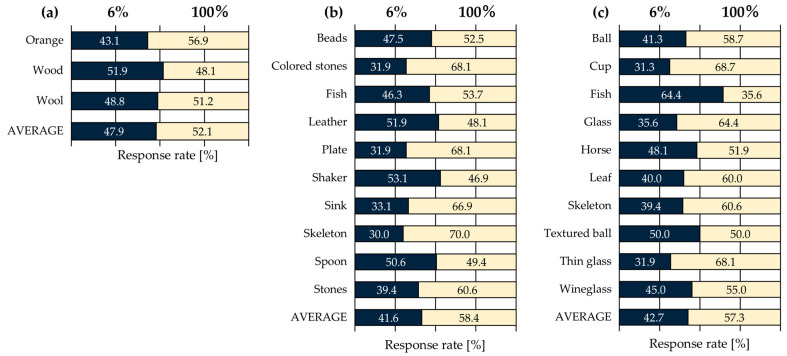
Average response rates of 10 observers: (**a**) roughness; (**b**) glossiness; (**c**) transparency.

The average response rates of the 10 observers who reported a stronger shitsukan perception for each attribute are shown in Figure 4. The average response rate was calculated as follows: for instance, if an observer selected the 100% pixel–aperture ratio five times and the 6% pixel–aperture ratio three times in a 6% vs. 100% pixel–aperture ratio pair, the response rate for the 100% pixel–aperture ratio was 5/8 (62.5%), while for the 6% pixel–aperture ratio, it was 3/8 (37.5%). The same calculation was performed for all ten observers, and the average response rate was determined based on the evaluation results for each pair (10 observers × 8 evaluations). Additionally, statistical *p*-values, standard deviations, effect sizes, and effect size indices were computed to assess whether differences in pixel–aperture ratio led to significant variations in shitsukan perception. In this study, a two-sample *t*-test was used as the inferential statistical test, and Cohen’s d was calculated as the measure of effect size. The closest classification was determined by Cohen and Sawilowsky [17,18]. For example, if the effect size was particularly large, it was classified as “Huge”, indicating a substantial perceptual difference between the stimulus pairs. This method enables the evaluation of effect size independently of the sample size.

Based on the results in Figure 4 and Table 1, focusing on the *p*-values, which indicate whether there is a significant difference between the average response rates, significant differences were observed in glossiness and transparency perception. Additionally, even when considering effect sizes independent of sample size, glossiness was classified as “Huge” and transparency as “Very Large”, confirming significant differences in these attributes. Furthermore, based on the relationship between the response rate and effect size, it was found that for all three shitsukan attributes, the 100% pixel–aperture ratio was perceived as exhibiting a stronger shitsukan perception compared to the 6% pixel–aperture ratio. These results indicate that differences in pixel–aperture ratio influence glossiness and transparency perception. However, these findings do not necessarily align with the superiority relationship of MTF values. A study by Shishikui et al. [19] reported that image resolution affects not only perceptual resolution but also lower-order visual impressions, such as color vividness, glossiness, and depth perception, with these impressions strengthening as resolution increases. Additionally, our previous study [10] found that higher MTF values corresponded to higher perceptual resolution. Based on these findings, we initially hypothesized that an increase in the MTF would enhance shitsukan perception. However, despite the 6% pixel–aperture ratio having a higher MTF than the 100% pixel–aperture ratio, observers reported a stronger shitsukan perception for the 100% pixel–aperture ratio. This inconsistency between the MTF and the evaluation results suggests that MTF alone cannot fully explain roughness, glossiness, and transparency perception. In previous studies analyzing the effects of sub-pixel arrays on shitsukan perception [8,9], the MTF superiority relationship was RGB > PenTile RGBG > RGBW. However, when examining the average response rates of all observers, roughness perception was rated as PenTile RGBG > RGB > RGBW, while transparency perception was rated as RGB > RGBW > PenTile RGBG, contradicting the expected MTF superiority relationship. These findings suggest that shitsukan perception is a complex phenomenon influenced not only by spatial frequency characteristics but also by pixel–aperture ratio and subjective observer evaluations. Additionally, individual differences among observers indicate that personal learning experiences and response tendencies to stimuli may have influenced their perception.

#### 3.2.2. Individual Differences

Next, we briefly introduce the results for individual observers. In roughness perception, no significant difference was observed in the average response rate. However, individual differences were evident. For instance, Observer 8 overwhelmingly favored the 6% pixel–aperture ratio (effect size: “Huge”), while Observer 10 strongly preferred the 100% pixel–aperture ratio (effect size: “Huge”). For glossiness perception, Observer 3 overwhelmingly favored the 100% pixel–aperture ratio (effect size: “Huge”), while Observer 6 also preferred the 100% pixel–aperture ratio (effect size: “Huge”). Similarly, in transparency perception, Observer 1 overwhelmingly favored the 6% pixel–aperture ratio (effect size: “Huge”), whereas Observer 10 strongly preferred the 100% pixel–aperture ratio (effect size: “Huge”).

These results suggest that differences in pixel–aperture ratio affect shitsukan perception. However, a consistent trend was not observed among individual observers, confirming large individual differences. For example, while some observers perceived stronger shitsukan perception with the 100% pixel–aperture ratio, others reported stronger shitsukan perception with the 6% pixel–aperture ratio. Such variations in individual evaluations may stem from subjective differences in perception among observers. Therefore, when assessing the influence of pixel–aperture ratio differences on shitsukan perception, it is essential to consider individual differences in perceptual characteristics and evaluation criteria.

### 3.3. Cluster Analysis: Observer Classification

**Figure 5:**
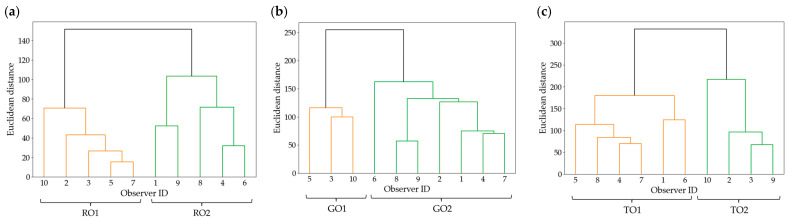
Dendrogram of observer cluster classification: (**a**) roughness; (**b**) glossiness; (**c**) transparency.

**Figure 6:**
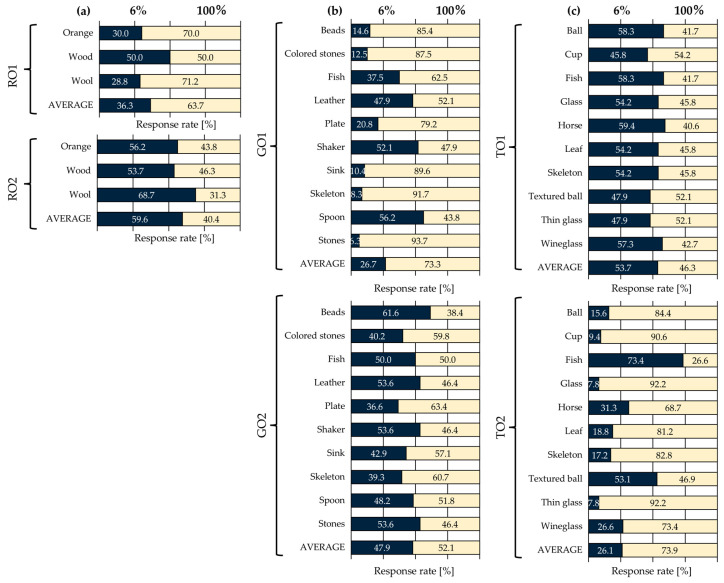
Response rates for each cluster: (**a**) roughness; (**b**) glossiness; (**c**) transparency.

Based on the results of the visual evaluation experiment, observers were classified using cluster analysis to account for individual response tendencies. Since each observer’s responses could exhibit distinct patterns, the goal of this analysis was to group observers based on their response data and clarify these differences. Hierarchical clustering (Ward’s method) was employed for this analysis. For clustering, a 46-dimensional response rate dataset per observer (23 different image contents × 2 pairs) was utilized. Figure 5 presents the dendrograms for (a) roughness perception, (b) glossiness perception, and (c) transparency perception. From Figure 5, it can be observed that all shitsukan attributes resulted in two clusters (RO1, RO2, GO1, GO2, TO1, TO2). Additionally, Figure 6 and Table 2 provide the average response rates for each cluster and the statistical significance indicated by effect sizes.

First, for roughness perception, Figure 5a confirms that RO1 and RO2 each consist of five observers. As shown in Figure 6 and Table 2, RO1 exhibited a preference for the 100% pixel–aperture ratio, while RO2 favored the 6% pixel–aperture ratio, leading to a clear division. Although no significant difference was observed in the *p*-values for either cluster, both exhibited a “Huge” effect size, indicating a substantial difference between the stimulus pairs. Regarding individual stimulus results, similar to previous studies [9], the greatest response rate difference was observed for the Wool stimulus, which contains a high concentration of high-frequency components. Since the roughness perception clusters were evenly split (five observers per group), this likely explains why no significant difference appeared in the overall average results.

Next, Figure 5b shows that GO1 consists of three observers, while GO2 includes seven. As shown in Figure 6 and Table 2, GO1 exhibited a preference for the 100% pixel–aperture ratio, with a statistically significant *p*-value and a “Huge” effect size, suggesting a substantial perceptual difference. GO2, however, did not show a significant *p*-value, and its effect size was classified as “Medium”, indicating no significant perceptual difference. These findings suggest that the 100% pixel–aperture ratio had a strong influence on glossiness perception but was not universally observed among all participants.

Finally, for transparency perception, Figure 5c shows that TO1 consists of six observers, while TO2 includes four. According to Figure 6 and Table 2, TO1 preferred the 6% pixel–aperture ratio, while TO2 favored the 100% pixel–aperture ratio. The *p*-values for both clusters indicated significant differences, with TO1 classified as “Very Large” and TO2 as “Huge”, confirming notable perceptual differences within each cluster.

In Section 3.2, the overall average response rates demonstrated a consistent relationship across shitsukan attributes, with the 100% pixel–aperture ratio > 6% pixel–aperture ratio. However, cluster analysis revealed that observers were divided into two distinct clusters for each shitsukan attribute, indicating that differences in pixel–aperture ratio led to variations in shitsukan perception, with notable individual differences among observers. Notably, in the cluster favoring the 100% pixel–aperture ratio, all effect sizes were classified as “Huge”, confirming its strong influence. Previous studies [8,9] also identified two or three observer clusters, showing different superiority relationships within each group. These findings suggest that individual differences play a critical role in the influence of pixel structure—including sub-pixel arrays and pixel–aperture ratios—on shitsukan perception.

### 3.4. Image Classification and Image Features

**Figure 7:**
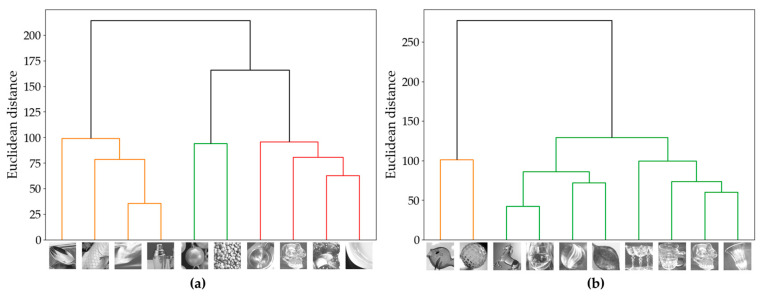
Dendrogram of observer cluster classification: (**a**) glossiness; (**b**) transparency.

**Figure 8:**
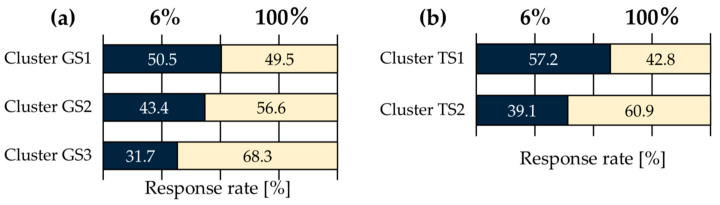
Average response rates for each stimulus cluster: (**a**) glossiness; (**b**) transparency.

**Figure 9:**
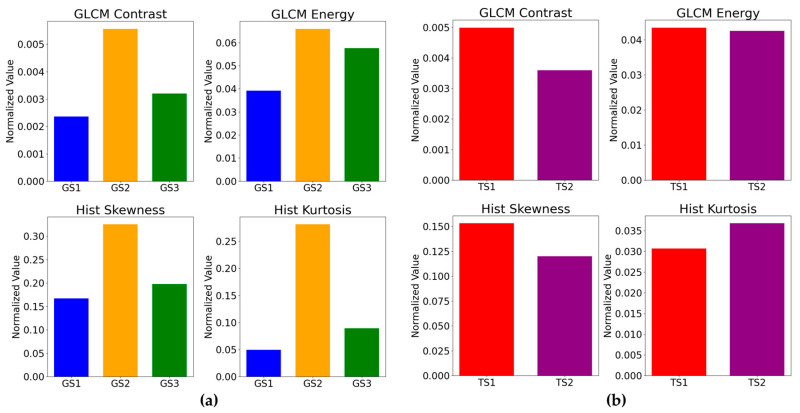
Image features for each stimulus: (**a**) glossiness; (**b**) transparency.

In addition to differences in pixel–aperture ratio, clustering was performed on ten stimuli for glossiness perception and transparency perception, and the feature values for each cluster were calculated to investigate which image characteristics influenced observer response tendencies. The image features used in this analysis included Contrast (an index of local luminance variation) and Energy (an index of texture repetition) from the gray level co-occurrence matrix (GLCM) [20], which is effective for texture analysis of objects in images, as well as kurtosis and skewness computed from the image luminance histogram. Kurtosis represents the sharpness or flatness of peaks, while skewness indicates the asymmetry of the distribution. These features are widely used to effectively capture texture characteristics [21]. The clustering results for glossiness perception and transparency perception are shown in Figure 7. Additionally, the response rates and effect sizes for each cluster are presented in Figure 8 and Table 3, while the average values of the computed image features for each cluster are shown in Figure 9. Each GLCM image feature is normalized to have a maximum value of 1. For glossiness perception, the stimuli were classified into three clusters. From left to right in Figure 7, these were named GS1 (spoon, fish, leather, shaker), GS2 (beads, stones), and GS3 (sink, skeleton, colored stones, plate).

#### 3.4.1. Glossiness Perception

First, we classified the glossiness perception clusters based on Hunter’s six types of gloss [8] as follows.

GS1(spoon, fish, leather, shaker): DOI gloss, low gloss

Among the GS1 group, the spoon and the shaker are characterized by clear reflection images, where glossiness is emphasized while maintaining a smooth and natural gloss perception. The image features of this group showed low Energy, skewness, and kurtosis, indicating that reflections are softly diffused in low gloss stimuli such as Fish and Leather. From Figure 8, it can be observed that the 6% pixel–aperture ratio was more frequently perceived as glossier. This suggests that the 6% pixel–aperture ratio may be better suited for the GS1 image group. This result can be attributed to the fact that a very high MTF leads to sharp-edge reproduction, enhancing DOI while maintaining soft reflection rendering.

GS2(beads, stones): contrast gloss, absence-of-bloom gloss

The GS2 image group is characterized by contrast gloss and absence-of-bloom gloss, with strong reflections and contrast in dark areas visually standing out, resulting in a glossiness perception where edges are sharply defined. The image features show high energy and contrast, with an asymmetric luminance distribution, making reflections more clearly visualized. From Figure 8, since the response rate was higher for the 100% pixel–aperture ratio, which can reproduce naturally smooth edges without excessive sharpness, it is suggested that the 100% pixel–aperture ratio may be more suitable despite a slight decrease in MTF.

GS3(sink, skeleton, colored stone, plate): specular gloss

The GS3 image group is characterized by specular gloss, with strong and distinct specular reflections particularly observed in Sink and Plate. This group exhibited relatively high Energy, emphasizing an overall strong and visually distinct gloss. Meanwhile, Contrast, skewness, and kurtosis were relatively low, meaning that the images included in this group tend to produce a more uniform gloss perception. From Figure 8, the 100% pixel–aperture ratio appeared more suitable for expressing specular gloss. The 100% pixel–aperture ratio allows smoother edge reproduction compared to the 6% pixel–aperture ratio, resulting in clear but not excessively sharp reflections. This leads to a visually natural and clear gloss perception, which was likely perceived as stronger. Previous studies [8] also reported that in clusters characterized by specular gloss, the response rate was higher for the PenTile RGBG sub-pixel array, which has a lower MTF, rather than the RGB sub-pixel array with a higher MTF. This suggests that for specular gloss, a moderate MTF may be more suitable than an excessively high MTF.

#### 3.4.2. Transparency Perception

For transparency perception, the stimuli were classified into two clusters. From left to right in Figure 7, these clusters were named TS1 (fish, textured ball) and TS2 (others). From Figure 8, TS1 was preferred with the 6% pixel–aperture ratio, while TS2 was preferred with the 100% pixel–aperture ratio. However, no significant differences were observed in terms of *p*-values or effect sizes. Looking at the image feature values, TS1 exhibited high Contrast, Energy, and skewness, while TS2 had high kurtosis. However, since TS2 included eight types of natural images, a direct comparison was difficult. Nevertheless, within TS1, the textured ball had the highest Contrast among all the natural images (value: 0.00541), and fish ranked fifth highest (value: 0.00457), indicating relatively high Contrast values.

This suggests that in cases where Contrast is high, the strong distinction between bright and dark areas and the well-defined edges may have contributed to a stronger perception of transparency with the 6% pixel–aperture ratio, which has a high MTF. However, as indicated by the TS2 results, the 100% pixel–aperture ratio was generally perceived as providing stronger transparency.

These results demonstrate that even for the same image stimuli, differences in pixel–aperture ratio can lead to significant variations in shitsukan perception. This discrepancy cannot be explained solely by the superiority of MTF values. Specifically, perceived shitsukan is influenced by both observer response tendencies and image content. Therefore, to achieve effective shitsukan management across various displays, in addition to MTF-focused methods [22], it is necessary to consider perceptual characteristics specific to each observer and image content, as shitsukan characteristics cannot be fully explained by MTF alone. In the development of shitsukan management technology, it will be important to take into account human visual characteristics, observer evaluation biases, and the specific content of images.

### 3.5. Discussion

In this study, stimuli were created using two types of pixel–aperture ratios (6% and 100%) with an RGB sub-pixel array, and their effects on the perception of three types of shitsukan were investigated. The MTF difference between these stimuli was approximately 17.4%, and it was confirmed that this difference had a statistically significant impact on shitsukan perception. However, a limitation of this study is that only two types of pixel structures with relatively high MTF values were used, and the sub-pixel array was fixed to the RGB configuration, restricting the scope of evaluation.

Furthermore, due to the constraints of the simulation in this study, only pixel–aperture ratios of 6% and 100% were examined. However, in actual displays, a 100% pixel–aperture ratio is rarely found. Recently, technological advancements in Micro-LED displays have led to the commercialization of displays achieving a black occupancy rate of over 99%, corresponding to a pixel–aperture ratio of 1% or less [23]. Therefore, under the conditions of this study, it is difficult to conclude that the influence of pixel–aperture ratio in real display environments was thoroughly examined.

To address this, the next section describes a supplementary experiment conducted to clarify the threshold at which humans can perceive differences in MTF.

## 4. Supplementary Experiment: MTF and Perceptual Resolution

In the supplementary experiment, a visual evaluation of perceptual resolution was conducted to clarify the threshold at which humans can perceive differences in MTF. The purpose was to comprehensively evaluate the influence of pixel–aperture ratio and MTF differences on perceptual resolution, not only by comparing high MTF values but also by including lower MTF values. Furthermore, by simplifying the experimental conditions and using vertical stripe stimuli instead of natural images, the criterion for the impact of MTF differences on perceptual resolution—rather than shitsukan perception—was clarified. Finally, based on the results from the additional experiment, we extrapolated how greater differences in pixel–aperture ratio may influence shitsukan perception.

### 4.1. Experimental Stimuli

**Figure 10:**
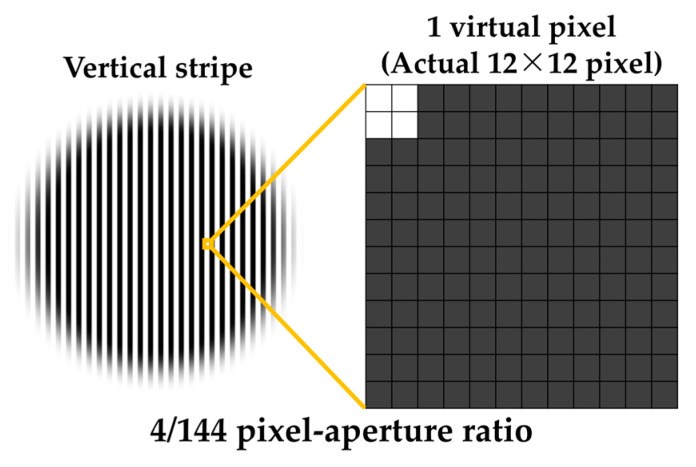
Construction of the virtual pixel structure for 4/144.

In the additional experiment, as outlined in Section 2.1, a 12 × 12 px region of the actual display was treated as a single virtual pixel. Eleven types of stimuli were created, simulating vertical stripe patterns with pixel–aperture ratios varied as 4/144, 9/144, 16/144, 25/144, 36/144, 49/144, 64/144, 81/144, 100/144, 121/144, and 144/144 (hereafter, pixel–aperture ratios are expressed in the form X/144 only). As an example, Figure 10 shows a schematic diagram of the experimental stimulus with a pixel–aperture ratio of 4/144. The other stimuli were generated by increasing the aperture area by one pixel in both the vertical and horizontal directions from the pattern in Figure 10. Due to simulation constraints, the sub-pixel array was not simulated in the additional experiment, and all aperture areas were set to white. To ensure consistency in the maximum luminance across stimuli, the white areas were set to 3.93 cd/m^2^. Each experimental stimulus consisted of a circular pattern with a diameter of 1800 px (150 virtual pixels), comprising 75 black-and-white vertical stripes. Additionally, to prevent the edges of the stimulus from serving as cues for responses, a gradient was applied to the outer edges of the circular stimulus.

### 4.2. Experimental Procedure

The additional experiment was conducted in a dark room with five observers whose binocular visual acuity was 1.0 or higher. Observers evaluated stimulus pairs displayed on the left and right sides of an LCD display (ColorEdge CG-248-4K, EIZO), selecting the stimulus in which the stripes appeared clearer using the 2AFC method. The stimulus pairs were designed to compare the following two conditions:

- The highest MTF condition (4/144) vs. all other pixel–aperture ratios (a total of 11 pairs).
- The lowest MTF condition (144/144) vs. all other pixel–aperture ratios (a total of 11 pairs).

These conditions allowed for a comparison of the effects of MTF differences on perceptual resolution in cases where the pixel–aperture ratio was extremely low or high.

The viewing distance was set to 30 cpd (5.65 m). To ensure reliability, each pair was evaluated 32 times. To counteract the effects of display luminance non-uniformity, the left and right positions of the stimulus pairs were swapped during the presentation. Throughout the entire experiment, each observer performed a total of 704 evaluations (11 pairs × 2 conditions × 32 evaluations per pair × 1 viewing distance).

### 4.3. Results and Discussions

**Figure 11:**
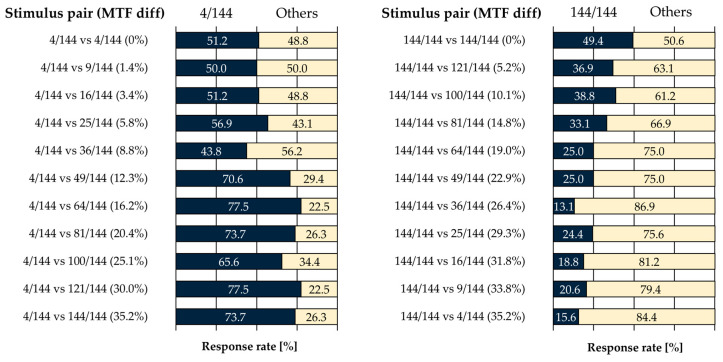
Average response rates of 5 observers.

Figure 11 shows the average response rates of the five observers who reported perceiving the striped pattern as clearer. Table 4 presents the corresponding effect sizes.

In the condition comparing a pixel–aperture ratio of 4/144 with other pixel–aperture ratios (Figure 11, left), no statistically significant difference was observed for stimulus pairs ranging from 4/144 vs. 4/144 to 4/144 vs. 36/144. The 4/144 vs. 4/144 condition involved identical stimuli on the left and right, and the absence of significant differences in both *p*-values and effect sizes indicates the stability of left–right responses in the evaluation task. Regarding effect sizes, only the pairs 4/144 vs. 25/144 and 4/144 vs. 36/144 were classified as “Very Large”, while all other pairs were categorized as either “Very Small” or “Small”. In contrast, for stimulus pairs from 4/144 vs. 49/144 to 4/144 vs. 144/144, statistically significant differences were observed, with effect sizes classified as “Huge”. These results suggest that, compared to the high MTF condition (4/144), an MTF difference of at least 12.3% is required for a significant perceptual distinction.

Next, in the condition comparing a pixel–aperture ratio of 144/144 with other pixel–aperture ratios (Figure 11, right), no statistically significant differences were observed in either *p*-values or effect sizes for the identical stimulus pair (144/144 vs. 144/144). However, for all other stimulus pairs, effect sizes were classified as “Huge”, and statistically significant differences were observed in all pairs except for 144/144 vs. 81/144. These results suggest that, compared to the low MTF condition (144/144), an MTF difference of at least 5.2% is required for a significant perceptual distinction in resolution.

Overall, the results demonstrate that for high MTF conditions, an MTF difference of 12.3% or more is necessary for a significant perceptual distinction, while for low MTF conditions, an MTF difference of 5.2% or more is sufficient. Considering the influence of pixel–aperture ratio on shitsukan perception, it is expected that displays with low pixel–aperture ratios will have a relatively minor impact on shitsukan perception. However, displays with high pixel–aperture ratios may experience a more pronounced effect, where even slight MTF differences—such as those caused by variations in sub-pixel arrays or pixel–aperture ratios—could significantly influence shitsukan perception.

In this study, we adopted a psychophysical experimental approach that emphasizes collecting a small number of high-quality data rather than increasing data quantity to improve statistical power. Although the number of participants was limited (10 in the paired stimulus experiment and 5 in the supplementary experiment), the reliability of the results was enhanced by the number of repeated evaluations. Specifically, in the first experiment, each participant made eight judgments for a stimulus pair, and in the supplementary experiment, each participant made 32 judgments for a single stimulus pair. In psychophysical paradigms, a response rate of 75% is often considered to reflect a statistically significant difference. As shown in Figure 6 and Figure 11, many stimulus pairs exceeded this threshold, supporting the interpretation that the observed differences are not merely due to random variation or outliers. To further demonstrate the robustness of our findings, we also reported effect sizes based on reliable average response rates obtained through a large number of evaluations. We acknowledge that standard effect size calculations typically rely on participant-level variability, and our approach differs in that the data are already stabilized through repeated measures. Nevertheless, the observation of large or very large effect sizes supports the conclusion that the observed effects are practically significant, even if statistical significance was not detected through *p*-values, which are known to be sensitive to small sample sizes. Increasing the number of participants would enhance the generalizability and statistical power of the findings.

The 2AFC method was used in this study, in which the observers were asked to select one of the stimuli. Alternatively, some designs introduce a neutral “neither” option when observers are unsure of their decision [24]. Providing such an option is considered useful to avoid forced or potentially inappropriate responses, and such alternative methods should be considered in the design of future experiments.

## 5. Conclusions

This study investigated the influence of a display’s pixel–aperture ratio on the perception of roughness, glossiness, and transparency through experimental analysis. Two types of pixel structures were used—6% and 100% pixel–aperture ratios—both featuring an RGB sub-pixel array. Based on the average response rates of 10 observers, the results revealed that glossiness and transparency perceptions were significantly stronger for the 100% pixel–aperture ratio compared to the 6% pixel–aperture ratio. In contrast, roughness perception exhibited substantial individual variability, with no significant difference in the average response rate. Furthermore, these trends did not necessarily align with the superiority of MTF values, indicating that visual shitsukan perception is influenced by a range of complex factors that go beyond the MTF alone.

Additionally, effect size calculations and cluster analysis identified two distinct clusters for each shitsukan attribute. In the cluster where the 100% pixel–aperture ratio predominated, all effect sizes were classified as “Huge”. This suggests that perceptual biases vary significantly among individuals, which in turn affects the evaluation of shitsukan. These findings highlight the importance of accounting for individual differences in display design. Furthermore, cluster analysis of the natural images used for glossiness and transparency evaluation, combined with image feature analysis, indicated that the suitability of either the 6% or 100% pixel–aperture ratio depends on the characteristics of the image group, particularly for glossiness perception. For transparency perception, although the 100% pixel–aperture ratio generally enhanced the perception of transparency, some images favored the 6% pixel–aperture ratio. These results demonstrate that the effect of pixel–aperture ratio on shitsukan perception is influenced not only by physical resolution or MTF differences but also by the image content and individual observer tendencies.

An additional experiment was conducted in this study to quantitatively assess the impact of MTF differences on perceptual resolution. The results confirmed that a perceptual difference became statistically significant when the MTF difference exceeded 12.3% in high MTF conditions and 5.2% in low MTF conditions. Applying these findings to shitsukan perception suggests that the impact on shitsukan perception is relatively minor when comparing displays with low pixel–aperture ratios. In contrast, when comparing displays with high pixel–aperture ratios, even slight changes in MTF can significantly affect shitsukan perception.

Future research needs to focus on further generalizing the effects of display pixel structure on shitsukan perception by analyzing a larger number of observers and a greater variety of image stimuli. For example, the effects of pixel aperture on shitsukan perception need to be investigated in more detail by preparing pre-sorted stimuli that are systematically categorized based on object type and Hunter’s six gloss categories. In addition, an important future research effort would be to quantify shitsukan perception based on image characteristics such as pixel aperture ratio, sub-pixel array, and the MTF. To achieve these goals, the sample size of experimental participants and image stimuli should be further expanded to investigate a wide variety of shitsukan perception and image characteristics. Another issue is to expand color images in consideration of real-life applications.

## Figures and Tables

**Table 1:** Effect sizes between stimulus pairs for all responses.

		Roughness	Glossiness	Transparency
	
Stimulus pair		6–100%	6–100%	6–100%
*p*-value		0.5012	0.0184	0.0438
Std. Dev.		4.43	9.29	9.87
Effect size		0.94	1.82	1.48
		Large	Huge	Very Large

**Table 2:** Effect sizes between stimulus pairs for different observer clusters.

		Roughness		Glossiness		Transparency	
	
Cluster		RO1	RO2	GO1	GO2	TO1	TO2
Stimulus pair		6–100%	6–100%	6–100%	6–100%	6–100%	6–100%
*p*-value		0.1839	0.1749	0.0046	0.4377	0.0391	0.0067
Std.Dev.		11.92	8.04	19.69	8.00	4.92	21.58
Effect size		2.31	2.38	2.37	0.51	1.53	2.22
		Huge	Huge	Huge	Medium	Very Large	Huge

**Table 3:** Effect sizes between stimulus pairs within each stimulus cluster.

		Glossiness	Transparency
	
Stimulus pair		6–100%	6–100%
*p*-value		0.2757	0.8701
Std.Dev.		9.47	12.82
Effect size		1.72	0.29
		Huge	Small

**Table 4:** Effect sizes between stimulus pairs for different observer clusters.

Stimulus pair	4/144-4/144	4/144-9/144	4/144-16/144	4/144-25/144	4/144-36/144	4/144-49/144	4/144-64/144	4/144-81/144	4/144-100/144	4/144-121/144	4/144-144/144
MTF diff	0.0	1.4	3.4	5.8	8.8	12.3	16.2	20.4	25.1	30.0	35.2
*p*-value	0.8033	1.0000	0.7572	0.2238	0.2488	0.0136	0.0049	0.0029	0.0469	0.0049	0.0029
Std.Dev.	10.50	7.33	8.44	10.69	10.36	10.96	10.92	8.15	12.30	10.92	8.15
Effect size	0.24	0.00	0.30	1.29	1.21	3.76	5.04	5.83	2.54	5.04	5.83
	Small	Very small	Small	Very large	Very large	Huge	Huge	Huge	Huge	Huge	Huge
Stimulus pair	144/144-144/144	144/144-121/144	144/144-100/144	144/144-81/144	144/144-64/144	144/144-49/144	144/144-36/144	144/144-25/144	144/144-16/144	144/144-9/144	144/144-4/144
MTF diff	0.0	5.2	10.1	14.8	19.0	22.9	26.4	29.3	31.8	33.8	35.2
*p*-value	0.8899	0.0196	0.0178	0.0667	0.0135	0.0025	0.0001	0.0088	0.0007	0.0019	0.0013
Std.Dev.	9.48	7.78	6.48	15.08	13.26	8.27	5.13	11.98	7.33	9.00	9.63
Effect size	0.13	3.37	3.47	2.24	3.77	6.05	14.36	4.28	8.53	6.53	7.14
	Small	Huge	Huge	Huge	Huge	Huge	Huge	Huge	Huge	Huge	Huge

## Data Availability

Data are available upon request due to restrictions.

## References

[1] Chadwick A.C., Kentridge R.W. (2015). The perception of gloss: A review. Vis. Res..

[2] Spence C. (2020). Shitsukan—the multisensory perception of quality. Multisens. Res..

[3] Bergmann Tiest W.M., Kappers A.M.L. (2007). Haptic and visual perception of roughness. Acta Psychol..

[4] Fleming R.W. (2014). Visual perception of materials and their properties. Vis. Res..

[5] Motoyoshi I., Nishida S., Sharan L., Adelson E.H. (2007). Image statistics and the perception of surface qualities. Nature.

[6] Fleming R.W., Bülthoff H.H. (2005). Low-level image cues in the perception of translucent materials. ACM Trans. Appl. Percept..

[7] Fleming R.W., Jäkel F., Maloney L.T. (2011). Visual perception of thick transparent materials: A model based on edge integration. Psychol. Sci..

[8] Tanaka M., Aketagawa K., Horiuchi T. (2024). Impact of display sub-pixel arrays on perceived gloss and transparency. J. Imaging.

[9] Aketagawa K., Tanaka M., Horiuchi T. (2024). Influence of display sub-pixel arrays on roughness appearance. J. Imaging Sci. Technol..

[10] Nakamura K., Tanaka M., Horiuchi T., Masaoka K. Effect of pixel aperture ratio on subjective spatial resolution. Proceedings of the International Display Workshops.

[11] Huang Y., Hsiang E.-L., Deng M.-Y., Wu S.-T. (2020). Mini-LED, Micro-LED and OLED displays: Present status and future perspectives. Light Sci. Appl..

[12] Lee T.-Y., Chen L.-Y., Lo Y.-Y., Swayamprabha S.S., Kumar A., Huang Y.-M., Chen S.-C., Zan H.-W., Chen F.-C., Horng R.-H. (2022). Technology and applications of micro-LEDs: Their characteristics, fabrication, advancement, and challenges. ACS Photonics.

[13] Sharan L., Rosenholtz R., Adelson E.H. (2014). Accuracy and speed of material categorization in real-world images. J. Vis..

[14] Tanaka M., Horiuchi T. (2015). Investigating Perceptual Qualities of Static Surface Appearance Using Real Materials and Displayed Images. Vis. Res..

[15] Hunter R.S. (1975). The Measurement of Appearance.

[16] Recommendation BT.2035. A Reference Viewing Environment for Evaluation of HDTV Program Material or Completed Programmes. ITU Radiocommunication Sector, 2013. https://www.itu.int/rec/R-REC-BT.2035/en.

[17] Cohen J. (1988). Statistical Power Analysis for the Behavioral Sciences.

[18] Sawilowsky S.S. (2009). New effect size rules of thumb. J. Mod. Appl. Stat. Methods.

[19] Shishikui Y., Sawahata Y. (2018). Effects of viewing ultra-high-resolution images with practical viewing distances on familiar impressions. IEEE Trans. Broadcast..

[20] Haralick R.M., Shanmugan K., Dinstein I. (1973). Textural Features for Image Classification. IEEE Trans. Syst. Man Cybern..

[21] Baran A., Piórkowski A., Strumiłło P., Klepaczko A., Strzelecki M., Bociąga D. (2024). Using histogram skewness and kurtosis features for detection of white matter hyperintensities in MRI images. The Latest Developments and Challenges in Biomedical Engineering.

[22] Tanaka M., Ando T., Horiuchi T. (2024). Automatic MTF conversion between different characteristics caused by imaging devices. J. Imaging.

[23] Sony Crystal LED.

[24] Kent M.G., Schiavon S. (2023). Predicting Window View Preferences Using the Environmental Information Criteria. LEUKOS.

